# Molecular characterization and antimicrobial susceptibility pattern of *Streptococcus agalactiae* isolated from clinical mastitis in dairy cattle

**DOI:** 10.1371/journal.pone.0199561

**Published:** 2018-06-21

**Authors:** Tiago Tomazi, Antonio Francisco de Souza Filho, Marcos Bryan Heinemann, Marcos Veiga dos Santos

**Affiliations:** 1 Department of Animal Production and Nutrition, Milk Quality Research Laboratory (Qualileite), University of São Paulo, Pirassununga, Brazil; 2 Department of Preventive Veterinary Medicine and Animal Health, Laboratory of Bacterial Zoonosis, University of São Paulo, São Paulo, Brazil; University of Illinois, UNITED STATES

## Abstract

The objectives of this study were to: (a) genotypically characterize *Streptococcus agalactiae* isolates recovered from clinical mastitis (CM) cases in dairy cows and, (b) determine the association of antimicrobial susceptibility (AMS) and genotypes of *Strep*. *agalactiae* clustered according to the genetic similarity. A total of 89 *Strep*. *agalactiae* isolates recovered from bovine CM were genotyped using random amplified polymorphic DNA (RAPD) analysis. In addition, the AMS of the isolates was determined using a commercial broth microdilution test composed of 10 antimicrobials (penicillin, ampicillin, oxacillin, cephalothin, ceftiofur, penicillin/novobiocin, erythromycin, pirlimycin, tetracycline, and sulfadimethoxine). Descriptive analysis was used to report the frequency of RAPD-types and genotypic clusters within herd, housing system, season and CM severity scores. The minimal antimicrobial concentrations that inhibited 50% (MIC_50_) and 90% (MIC_90_) of the isolates were calculated and survival analysis was completed to verify the differences of AMS among genotypic clusters. Results of RAPD showed a great genotypic diversity of *Strep*. *agalactiae* (45 RAPD-types) and three clusters (Ia, Ib and II) were created based on the genetic similarity among genotypes. After clustering, a high genetic similarity was observed within and between herds. Overall, *Strep*. *agalactiae* showed high susceptibility to most antimicrobials, except to tetracycline and erythromycin. Differences in the AMS among clusters were observed for ampicillin, ceftiofur, erythromycin, pirlimycin, sulfadimethoxine and tetracycline. In conclusion, *Strep*. *agalactiae* is still highly susceptible to most antimicrobials, although differences in susceptibility to certain antimicrobials were observed among genotypic clusters.

## Introduction

*Streptococcus agalactiae* is a contagious pathogen of bovine mastitis, and the mammary gland is considered as the main reservoir of this bacteria in dairy herds. Transmission of *Strep*. *agalactiae* occurs mainly from cow-to-cow via milking units, liners, milkers’ hands or towels of common use [1]. The prevalence of *Strep*. *agalactiae* have become very rare in herds of North America and in some regions in Europe [2–4], especially because of specific control programs aiming to reduce contagious mastitis. However, *Strep*. *agalactiae* is still an important cause of bovine intramammary infection (IMI) in other regions, such as certain countries in South America. For example, in studies conducted in dairy herds of Colombia, the prevalence of cows with IMI caused by *Strep*. *agalactiae* varied from 28 to 35% [5, 6]; in Brazil, a recent study of our research group reported this pathogen as the third most prevalent bacteria causing bovine CM in 20 dairy herds [7]. Furthermore, a reemergence of *Strep*. *agalactiae* has been described in northern Europe [8, 9], where nonbovine sources (i.e., people) were reported to be the main cause of reintroduction of this pathogen in dairy herds [10, 11].

Genotyping methods can contribute to advancing mastitis epidemiological understanding by the identification of strains and their specific characteristics, such as potential reservoirs, route of transmission, virulence factors, pathogenicity, and antimicrobial susceptibility (AMS) [12]. Molecular methods have been successfully used in studies characterizing the epidemiology of *Strep*. *agalactiae* causing bovine mastitis, which includes pulsed-field gel electrophoresis (PFGE) technique [13, 14], restriction fragment length polymorphism (RFLP) PCR [15], multilocus sequence typing (MLST) [16], and whole-genome sequencing [17]. In addition, random amplified polymorphic DNA (RAPD) PCR was used effectively in epidemiological studies evaluating the genotypic diversity of *Strep*. *agalactiae* [18]. Some of the advantages of RAPD-PCR over other typing methods are the simplicity of procedure and low cost, while the major disadvantage is the inability to compare RAPD-types between studies, as the differentiation of strains is based on DNA fragment distribution on the agarose gel. However, this method has sufficient discriminatory power and acceptable reproducibility to characterize the genotypic diversity of *Streptococcus* spp. isolated from IMI in different herds, especially when the isolates are analyzed simultaneously [19].

Molecular epidemiology of *Strep*. *agalactiae* causing CM was recently reported in some countries, such as Colombia [20], China [21], Denmark [14] and Poland [22]. In addition, a molecular characterization of *Strep*. *agalactiae* recovered from milk samples of dairy cows in Brazil was performed using MLST, genotypic capsular typing by multiplex PCR, and virulence gene detection; however, few isolates (12 of 59) were identified from CM cases, while the remaining were from subclinical mastitis [16]. Furthermore, a recent study reported frequency differences of dominant strains of *Strep*. *agalactiae* between countries and continents [20], indicating that results from one country do not necessarily represent the situation in a different country. Therefore, considering that *Strep*. *agalactiae* is still an important cause of clinical mastitis in dairy herds of Brazil, the genotypic characterization of this species can advance the epidemiological understanding of *Strep*. *agalactiae* causing CM and, consequently, improve programs aiming to control this pathogen in dairy herds.

Although prevention strategies such as adoption of good milking procedures and within-herd biosecurity programs can reduce the risk of new IMI caused by *Strep*. *agalactiae*, they do not eliminate these pathogens from the herd, especially in scenarios of high prevalence of chronically infected cows. Therefore, antimicrobial therapy remains the main strategy for the control of this bacteria in dairy herds [1]. However, the non-judicious use of antimicrobials for treatment of mastitis in dairy cattle has been associated with increased risk of antimicrobial resistance in both veterinary and human medicine [23, 24]. Furthermore, resistant strains of *Strep*. *agalactiae* causing bovine mastitis have been reported [13, 25].

Besides the conscious use of antimicrobials for mastitis treatment, monitoring the AMS of bacteria causing bovine mastitis is an essential component of prevention programs for reducing the proliferation of resistant strains. The monitoring of antimicrobial resistance among *Strep*. *agalactiae* isolated from CM may help veterinarians and pharmaceutical industries improve control and treatment strategies of this pathogen in dairy herds [22]. To our knowledge, only one recent study reported the molecular epidemiology of *Strep*. *agalactiae* causing IMI in dairy herds of Brazil. However, a limited number of isolates were recovered from CM [16] and, in addition, the evaluation of AMS of *Strep*. *agalactiae* causing bovine CM was not performed in that study. Hence, the objectives of this study were to genotypically characterize *Strep*. *agalactiae* isolates causing CM in dairy cows and to determine the association of AMS and genotypes of *Strep*. *agalactiae* clustered according to the genetic similarity.

## Material and methods

### Ethics statement

The study was approved by the Ethics Committee on Animal Use of the School of Veterinary Medicine and Animal Science of University of São Paulo (registration code: CEUA 2994060214). All experimental procedures and the care of cows were in strict accordance with the rules issued by the Brazilian National Council for Control of Animal Experimentation (CONCEA; Law 11.794 of October 8, 2008, Decree 6899 of July 15, 2009).

### Bacterial isolates

Isolates were selected from a collection of *Strep*. *agalactiae* identified during an epidemiological study on characterization of CM, in which 248 *Strep*. *agalactiae* were identified after culture of 4,212 individual quarter milk samples collected in 20 dairy herds from 2,637 cows with CM [7]. Bacteriological identification was performed according to recommendations of National Mastitis Council [26]. Briefly, a 0.01 mL sterile loop was used to plate milk samples onto trypticase soy agar (TSA) plates (BBL-Becton Dickinson and Co., LePoint de Claix, France) enriched with 5% bovine blood, and plates were incubated aerobically at 37°C. Phenotypic features were examined at 24 and 48 h after incubation and specific biochemical testing was performed to determine bacterial genus and/or species. *Streptococcus agalactiae* isolates were identified as Gram-positive cocci, with negative reaction for catalase and esculin activity, and positive Christie-Atkins-Munch-Peterson (CAMP) reaction.

Descriptive data reporting the frequency of identified *Strep*. *agalactiae* from cases of CM in the aforementioned study [7], as well as, the frequencies of cryopreserved, re-cultured, and selected isolates for the current study are presented in Table 1. Out of 248 isolates identified during bacteriological culture, 48 isolates were not cryopreserved because of one of the following reasons: (a) identified from a CM case occurring in the same quarter of the same cow within 14 days after the first case (i.e., not considered as new case of CM; n = 40); and (b) identified from mixed cultures (more than one pathogen isolated in the same culture; n = 8). Furthermore, before selection of isolates for this study, all cryopreserved bacteria were thawed and re-cultured for species confirmation [26], and only isolates identified as *Strep*. *agalactiae* from pure cultures were selected.

**Table 1:** Descriptive herd-level data, frequency of CM (overall and pathogen-specific), and frequency of *Strep*. *agalactiae* isolates that were cryopreserved, re-cultured, and selected for genotyping assessment and antimicrobial susceptibility testing.

Herd	Size^1^	Period in the study	Freq. CM^2^	*SA* cases^3^	*SA cryopres*.^4^	*SA* recult.^5^	*SA* selected^6^
B	184 (25)	Apr/14 –Apr/15	179	2	2	1	1
D	165 (11)	Apr/14 –Apr/15	225	1	-	-	-
E	371 (24)	Apr/14 –Apr/15	627	1	1	-	-
G	77 (15)	Apr/14 –Nov/14	43	4	4	4	4
I	167 (11)	May/14 –Apr/15	383	30	25	19	13
J	120 (10)	Apr/14 –Apr/15	72	1	1	1	1
K	313 (7)	Mar/14 –Apr/15	314	7	7	4	4
M	586 (17)	Dec/14 –Dec/15	1,395	26	16	9	9
N	55 (7)	Apr/14 –Jun/15	112	41	33	28	20
P	36 (1)	Apr/14 –Apr/15	69	1	-	-	-
Q	75 (12)	May/14 –Apr/15	220	132	110	78	36
S	55 (7)	Jun/14 –May/15	52	2	1	1	1
Overall	184 (164)	Mar/14 –Jun/15	3,691	248	200	145	89

^1^Herd size–Average number of lactating cows (standard deviation in parenthesis) per herd during the study period.

^2^Frequency (n) of clinical mastitis cases identified during the study period regardless of causing-pathogen.

^3^Frequency (n) of clinical mastitis cases with identification of *Streptococcus agalactiae* (*SA*) in the bacteriological culture.

^4^Frequency (n) of isolates cryopreserved during the study period.

^5^Frequency (n) of isolates from pure cultures (without contamination) during bacteriological culture performed before selection.

^6^Frequency (n) of isolates selected for genotyping assessment and antimicrobial susceptibility testing.

A total of 145 isolates presented pure bacterial growth during re-culturing performed before molecular testing, however only 89 *Strep*. *agalactiae* were selected for this study. The number of isolates was established because we aimed to genotype all isolates in a single batch of RAPD-PCR to avoid potential variation of the results that could happen if isolates were segregated in more than one batch on analysis [27]. Selected isolates were recovered from milk samples of 62 dairy cows diagnosed with CM in 9 dairy herds (Table 1). A total of 46 isolates were identified from 19 cows that had repeated cases of CM: 11 cows had isolates from two cases, and 8 cows had isolates from three repeated cases of CM.

Selection of isolates was performed with the intention to include in the study strains identified in all herds. Thus, all re-cultured isolates from herds with less than 15 isolates that attended the inclusion criteria were selected for this study; whereas, for herds with ≥15 isolates, strains were randomly selected using the RAND function of Excel software (2010; Microsoft Office Corporation, Redmond, WA, USA).

### DNA extraction and RAPD typing

Extraction of genomic DNA from pure cultures (i.e., no contamination) was performed with the illustra bacteria genomicPrep Mini Spin Kit (GE Healthcare, United Kingdom) following the manufacturer’s guidelines.

### RAPD typing

The amplification of the bacterial DNA was performed as described by Martinez et al. [18], with some modifications. Briefly, the PCR mixture consisted of 12.5 μL of Go Taq® Green Master Mix, 2X (Promega, Madison, USA), 1.25 μL of primer OPB-17 (5’-AGGGAACGA-3’; Exxtend Solution in Oligos, Campinas, Brazil), 5 μL of genomic DNA (20 ng/μL), and water qsp to a final volume of 25 μL. The mixture was then submitted for thermocycling using the following program: one initial cycle of denaturation (5 min at 94°C) in a DNA Thermal Cycler (Eppendorf Mastercycler^®^ Gradient, Hamburg, Germany); and 44 subsequent cycles consisting of denaturing at 94°C for 30 s, annealing at 35°C for 1 min, and extension at 72°C for 5 min. Ramp times were at 0.5°C/s. A negative control, consisting of the same reaction mixture but with water instead of DNA, was included in the run. In addition, a positive control, with a template of DNA from a reference strain (*Strep*. *agalactiae* ATCC 13813), was also amplified.

All amplified products were electrophoresed in a single batch in 1.5% agarose gel with 96 wells using Tris-borate-EDTA buffer (TBE; pH 8.3) at 150 V for 180 min. The agarose gel was stained with *SYBR™ Safe DNA Gel Stain* (1:10,000; Thermo Scientific^®^, Carlsbad, USA). Images of gel were taken under ultraviolet light using a photo-documentation system (Syngene, GeneGenius, Cambridge, United Kingdom). Band sizes were determined by comparison to a 100-bp DNA ladder (Promega, Madison, WI, USA).

### Antimicrobial susceptibility testing

A mastitis panel of a commercial broth microdilution test (CMV1AMAF; Sensititre^®^, TREK Diagnostic Systems, LLC, Cleveland, OH, USA), was used for AMS testing following guidelines of the Clinical and Laboratory Standards Institute (CLSI, [28, 29], formerly NCCLS). The following antimicrobial agents with their dilution ranges were tested: penicillin (0.12–8.0 μg/mL), ampicillin (0.12–8.0 μg/mL), oxacillin (2.0–4.0 μg/mL), cephalothin (2.0–16.0 μg/mL), ceftiofur (0.5–4.0 μg/mL), penicillin/novobiocin (1.0/2.0–8.0/16.0 μg/mL), erythromycin (0.25–4.0 μg/mL), pirlimycin (0.5–4.0 μg/mL), tetracycline (1.0–8.0 μg/mL), and sulfadimethoxine (32.0–256.0 μg/mL).

Briefly, cryopreserved isolates were thawed, inoculated on blood agar and incubated at 37°C for 24 h. Afterward, a single and pure colony was re-inoculated on Trypticase Soy Agar (TSA; BD, Sparks, MD, USA) and incubated at 37°C for 24 h. After incubation, the isolates were suspended in 0.9% saline solution using disposable sterile loops to approximate the density of a 0.5 McFarland standard. A DEN-1 McFarland Densitometer (Biosan, Riga, Latvia) was used for standardization of bacterial suspensions to the McFarland scale. Subsequently, 100 μL-aliquots of bacteria suspensions were transferred into a tube containing 11 mL of Mueller-Hinton broth (pH = 7.3 ± 1; BD, Sparks, MD, USA) supplemented with 5% of lysed horse blood, and the tube was mixed on a vortex for approximately 10 s.

Sensititre^®^ panels were reconstituted with the bacteria inoculum (50 μL per wheel) using a multichannel pipette with disposable tips. Panels were covered with an adhesive seal and incubated at 35°C for approximately 20–24 h. After incubation, results were read using the Sensititre® manual viewer (TREK Diagnostic Systems, LLC, Cleveland, OH, USA). Growth appeared as turbidity or as a deposit of cells at the bottom of a well. The MIC was recorded as the lowest concentration of the antimicrobial that inhibited visible growth. Control wells were evaluated first, and if any did not exhibit growth, the results were considered invalid. For each batch of microorganism tested, the NCCLS recommended quality control strains *Escherichia coli* ATCC 25922 and *Staph*. *aureus* ATCC 29213 were also evaluated.

### Data analysis

The FREQ procedure of SAS 9.4 (SAS Institute Inc., Cary, NC, USA) was used to determine the distribution of RAPD-types within herd, season, housing system, and severity score of CM; data were expressed as absolute numbers and percentages. For descriptive analysis, the bacterial isolates were classified in categories based on: herd from which the isolate was identified; CM severity (**Mild**—changes only in the milk appearance; **Moderate**—presence of abnormal milk accompanied by changes in the udder; or, **Severe**—combination of abnormal milk, with signs of inflammation in the udder and systemic signs); season of CM diagnosis (**rainy—**October-March, or **dry—**April-September; Oliveira et al., [30]); and housing system (compost-bedded pack barn, free stall, paddocks). The paddock housing system was characterized as an open area surrounded by fences or rails and without pasture for grazing.

For RAPD fingerprinting results, a dendrogram based on the intraspecific diversity and genetic relationship of strains were constructed using BioNumerics software v. 6.6 (Applied Maths, Sint-Martens-Latem, Belgium). The dendrograms was generated by the unweighted pair group method with arithmetic mean (UPGMA) using the default configuration with both position tolerance and optimization of 1%.

The AMS testing results were evaluated based on the minimal concentrations that inhibited 50% (MIC_50_) and 90% (MIC_90_) of the isolates’ growth. Where interpretive cut-points for MIC of the evaluated antimicrobials have been established for *Strep*. *agalactiae* [28, 29], these were used to classify each isolate as susceptible, intermediate or resistant. Isolates identified as intermediate were interpreted as resistant.

Clusters were created based on genetic similarity of isolates according to the dendrogram construction. Survival analysis using the PROC LIFETEST of SAS (SAS Institute Inc., Cary, NC, USA) was completed to verify the differences between the AMS among genotypic clusters [31]. The concentrations of antimicrobials contained in the commercial broth microdilution test were used as the “time” variable, and the inhibition of bacterial growth was used as the event. Isolates that presented growth at the highest tested concentration were censored by the statistical model. Kaplan-Meier survival curves of the RAPD-types clustered based on their genetic relationship were performed for each antimicrobial studied. The null hypothesis of no differences (homogeneity of survival curves) in the survivor functions of the strata (RAPD-clusters) was evaluated using Log-Rank and Wilcoxon tests. Statistical significance (i.e., heterogeneous survival curves) was defined at *P* ≤ 0.05.

## Results

### Descriptive results and RAPD typing

**Fig 1:**
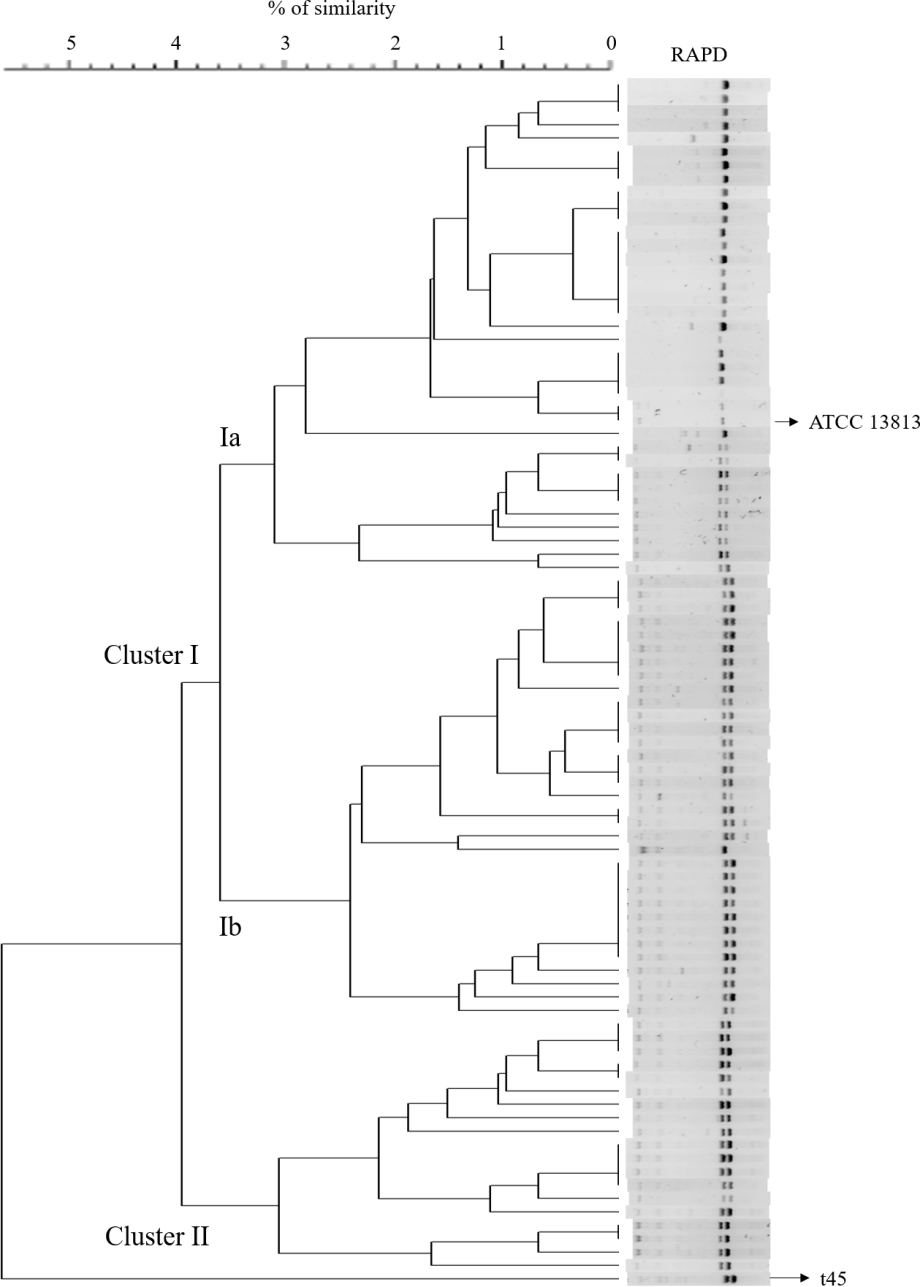
Dendrogram of RAPD profiles of 89 *Streptococcus agalactiae* isolated from bovine clinical mastitis in 9 dairy herds of southeastern Brazil. Three clusters were created based on the genetic relationship of isolates (Ia, n = 36; Ib, n = 33; and II, n = 19). One isolate (t45) was not included into clusters because it had a lower level of similarity in comparison to other isolates. The dendrogram was generated by the unweighted pair group method with arithmetic averages.

After genotyping, a high level of polymorphism was observed among isolates (45 RAPD-types) and the genotypes were clustered into two groups according to their genetic similarity (clusters I and II; Fig 1). One isolate (named here as t45) was not included into clusters because it had a lower level of similarity in comparison to other isolates (Fig 1). Of the 88 remaining isolates, 69 (78.4%) belonged to cluster I, which was composed of 32 RAPD-types, while 19 isolates (21.3%) belonged to cluster II (12 RAPD-types). For statistical purposes and based on the genetic similarity of isolates within group, cluster I was divided into two subgroups (Ia and Ib); therefore, cluster Ia had 36 isolates (18 RAPD-types), and cluster Ib had 33 isolates (12 RAPD-types; Fig 1; Table 2).

**Table 2:** Distribution of 89 *Streptococcus agalactiae* isolates from CM cases occurred in 9 dairy herds of southeastern Brazil according to RAPD-clusters (Ia [n = 36], Ib [n = 33], and II [n = 19]), herd of origin, housing system, season and severity score of clinical mastitis.

Variable	Categories	Frequency (n) isolates	Frequency (n) RAPD-types	RAPD-clusters						t 45	
				Ia		Ib		II			
				n	%	n	%	n	%	n	%
Herd	B	1	1	1	100	-	-	-	-	-	-
	G	4	3	-	-	-	-	4	100	-	-
	I	13	10	8	61.6	-	-	5	38.4	-	-
	J	1	1	-	-	-	-	1	100	-	-
	K	4	2	4	100	-	-	-	-	-	-
	M	9	7	2	22.2	-	-	7	77.8	-	-
	N	20	8	20	100	-	-	-	-	-	-
	Q	36	17	-	-	33	91.7	2	5.6	1	2.7
	S	1	1	1	100	-	-	-	-	-	-
Housing	Freestall	33	17	26	78.8	-	-	7	21.2	-	-
	CBPB^1^	51	29	9	17.6	33	64.7	8	15.7	1	2.0
	Paddocks	5	4	1	20.0	-	-	4	80.0	-	-
Season^2^	Rainy	46	29	16	34.8	19	41.3	10	21.7	1	2.2
	Dry	43	26	20	46.5	14	32.6	9	20.9	-	-
Severity^3^	Mild	59	36	20	33.9	23	39.0	16	27.1	-	-
	Moderate	20	14	9	45.0	9	45.0	1	5.0	1	5.0
	Severe	5	4	5	100.0	-	-	-	-	-	-

^1^ CBPB–Compost bedded pack barn.

^2^ Rainy season (October-March), Dry season (April-September; Oliveira et al., [30]).

^3^ Severity of clinical mastitis—(Mild) changes only in the milk appearance; (Moderate) presence of abnormal milk accompanied by changes in the udder; or, (Severe) combination of abnormal milk, with signs of inflammation in the udder and systemic signs).

A total of 6 farms (B, G, J, K, N and S) had isolates belonging to a unique cluster, while three farms (I, M and Q) had isolates from more than one cluster. Isolates belonging to cluster Ia were identified in six of nine herds, while all isolates belonging to cluster Ib derived from the same farm (herd Q). In addition, isolates belonging to cluster II were identified in five herds (Table 2).

Of cows with repeated cases of CM (n = 19), seven had identification of the same RAPD-type identified in the first case regardless of affected mammary quarter; whereas the remaining 12 cows had different RAPD-type identified in the second or third cases of CM compared to the first case. Eight cows had repeated cases of CM in the same quarter, but only two of these cows had the same RAPD-type as identified in the previous CM case (all strains belonged to cluster Ib); for the other six cows, a different RAPD-type was identified, although all strains isolated within cows belonged to the same cluster (four cows had isolates belonging to cluster Ib, and two cows had isolates belonging to cluster Ia).

A total of 33 isolates were identified from herds housing cows in free stall (26 belonged to cluster Ia, and 7 to cluster II), while 51 isolates were from herds with CBPB system (9 belonged to cluster Ia, 33 to cluster Ib, and, 8 to cluster II). Furthermore, 5 isolates were from herds with paddock housing system (1 isolate belonged to cluster Ia, and the other 4 were assigned to cluster II; Table 2).

In relation to the frequency of *Strep*. *agalactiae* according to the season, 46 (51.7%) isolates were identified from CM cases occurring in the rainy season. Of these, 16 (34.8%) belonged to cluster Ia, 19 (41.3%) to cluster Ib, and 10 (21.7%) to cluster II. For the isolates identified during the dry season (n = 43), 20 (46.5%) belonged to cluster Ia, 14 (32.6%) to cluster Ib, and 9 (20.9%) to cluster II (Table 2).

A total of 59 (70.2%) *Strep*. *agalactiae* isolates were recorded as causing mild CM cases, while 20 (23.8%) were associated with moderate, and 5 (6.0%) with severe CM cases. For those isolates identified from mild CM cases, 20 (33.9%) isolates belonged to cluster Ia, 23 (39.0%) to cluster Ib, and 16 (27.1%) to cluster II. For isolates identified in moderate CM cases, 9 (45.0%) belonged to cluster Ia, 9 (45.0%) to cluster Ib, and 1 (5.0%) to cluster II. Finally, all isolates identified from severe CM cases (n = 5) belonged to cluster Ia (Table 2).

### Antimicrobial susceptibility testing

#### Overall evaluation

A total of 86 (96.6%) *Strep*. *agalactiae* isolates had results of AMS. Two isolates were excluded (postadmission) from data analysis of AMS because of contamination during microdilution testing. In addition, AMS results of the isolate identified as “t45”, which was not clustered into groups I or II, was not included in data analysis of AMS. For the other 86 isolates, overall results on the AMS are shown in Tables 3 and 4. Isolates showed high susceptibility to ampicillin (98.3%), ceftiofur (97.7%), cephalothin (96.5%), oxacillin (96.5%), penicillin+novobiocin (100%), penicillin (97.7%), pirlimycin (83.7%) and sulfadimethoxine (98.3%). On the other hand, lower susceptibility frequencies were observed for erythromycin (70.9%) and tetracycline (31.4%).

**Table 3:** Frequency (number, and percentages in parenthesis) of *Streptococcus agalactiae* classified as susceptible (S) or resistant (R), according to the interpretation criteria described by the Clinical and Laboratory Standards Institute (CLSI, [28, 29]).

Antimicrobial	CLSI criteria^1^	RAPD-clusters			Overall
		Ia (n = 35)	Ib (n = 32)	II (n = 19)	
Ampicillin	R	-	-	1 (5.3)	1 (1.7)
	S	35 (100)	32 (100)	18 (94.7)	85 (98.3)
Ceftiofur	R	2 (5.7)	-	-	2 (2.3)
	S	33 (94.3)	32 (100)	19 (100)	84 (97.7)
Cephalothin	R	-	2 (6.2)	1 (5.3)	3 (3.5)
	S	35 (100)	30 (93.8)	18 (94.7)	83 (96.5)
Erythromycin	R	1 (2.9)	22 (68.7)	2 (10.6)	25 (29.1)
	S	34 (97.1)	10 (31.3)	17 (89.4)	61 (70.9)
Oxacillin	R	-	2 (6.2)	1 (5.3)	3 (3.5)
	S	35 (100)	30 (93.8)	18 (94.7)	83 (96.5)
Penicillin+Novobiocin	R	-	-	-	-
	S	35 (100)	32 (100)	19 (100)	86 (100)
Penicillin	R	2 (5.7)	-	-	2 (2.3)
	S	33 (94.3)	32 (100)	19 (100)	84 (97.7)
Pirlimycin	R	-	14 (43.7)	-	14 (16.3)
	S	35 (100)	18 (56.3)	19 (100)	72 (83.7)
Sulfadimethoxine	R	1 (2.9)	-	-	1 (1.7)
	S	34 (97.1)	32 (100)	19 (100)	85 (98.3)
Tetracycline	R	26 (74.3)	30 (93.7)	3 (15.8)	59 (68.6)
	S	9 (25.7)	2 (6.3)	16 (84.2)	27 (31.4)

^1^S = susceptible

R = resistant.

**Table 4:** Overall frequency (%) of *Streptococcus agalactiae* isolates (n = 86) that had 50% (MIC_50_) and 90% (MIC_90_) of bacterial growth inhibited at each antimicrobial concentration. All isolates were identified from clinical mastitis cases occurred in 9 dairy herds of Southeast, Brazil.

Antimicrobial	Frequency (%) of isolates at each indicated MIC (μg/mL)^1^												NI^2^	MIC_50_^3^	MIC_90_^4^
	0.12	0.25	0.5	1	2	4	8	16	32	64	128	256			
Ampicillin	67.8	31.1	1.1	0.0	0.0	0.0	0.0	-	-	-	-	-	-	0.12	0.25
Ceftiofur	-	-	87.5	9.2	1.1	1.1	-	-	-	-	-	-	1.1	0.5	1
Cephalothin	-	-	-	-	96.6	2.3	0.0	0.0	-	-	-	-	1.1	2	2
Erythromycin	-	71.3	5.7	5.7	3.5	4.6	-	-	-	-	-	-	9.2	0.25	4
Oxacillin	-	-	-	-	96.6	1.1	-	-	-	-	-	-	2.3	2	2
Penicillin+Novobiocin	-	-	-	100.0	0.0	0.0	0.0	-	-	-	-	-	-	1	1
Penicillin	97.7	2.3	0.0	0.0	0.0	0.0	0.0	-	-	-	-	-	-	0.12	0.12
Pirlimycin	-	-	82.9	1.1	0.0	1.1	-	-	-	-	-	-	14.9	0.5	>4
Sulfadimethoxine	-	-	-	-	-	-	-	-	23.0	46.0	23.0	6.9	1.1	64	256
Tetracycline	-	-	-	21.8	9.2	2.4	1.1	-	-	-	-	-	65.5	>8	>8

^1^The light gray shading represents the susceptible zone, and the darker gray shading represents the resistant zone. Results were interpreted according to the Clinical and Laboratory Standards Institute (CLSI, [28, 29]). Interpretative criteria were based on human data (ampicillin, erythromycin, oxacillin, penicillin, sulfadimethoxine and tetracycline), dogs’ data (cephalothin), and bovine mastitis (ceftiofur, penicillin/novobiocin and pirlimycin). The resistant category included those isolates categorized as either intermediate or resistant.

^2^NI = Not inhibited (growth at highest concentration tested).

^3^MIC (μg/mL) that inhibited 50% (MIC_50_) of the isolates.

^4^MIC (μg/mL) that inhibited 90% (MIC_90_) of the isolates.

For all antimicrobials, except two (sulfadimethoxine and tetracycline), the MIC_50_ was observed at the lowest antimicrobial concentration contained in the microdilution test (Table 3). The MIC_50_ for sulfadimethoxine was 64 μg/mL, while more than 50% of the isolates were not inhibited at the highest tetracycline concentration contained in the microdilution test. The antimicrobial concentration needed to inhibit 90% of all tested isolates were: ampicillin (0.25 μg/mL), ceftiofur (1 μg/mL), cephalothin (2 μg/mL), erythromycin (4 μg/mL), oxacillin (2 μg/mL), penicillin+novobiocin (1 μg/mL), penicillin (0.12 μg/mL), and sulfadimethoxine (256 μg/mL). For both pirlimycin and tetracycline, more than 10% of isolates had growth at the highest antimicrobial concentration present in the microdilution panel, and the MIC_90_ was not estimated (Table 4).

#### Cluster evaluation

When *Strep*. *agalactiae* isolates were classified according to RAPD-clusters, differences were observed in the AMS among them. For example, 97.1% of isolates belonging to cluster Ia and 89.4% of isolates belonging to cluster II were susceptible to erythromycin, while a lower frequency (31.3%) of isolates belonging to cluster Ib was susceptible to the same antimicrobial (Table 5). Furthermore, although 100% of isolates belonging to clusters Ia and II were classified as susceptible to pirlimycin, only 53.2% of isolates assigned to cluster Ib were susceptible to the same antimicrobial (Table 6). For tetracycline, the susceptibility was 6.2% for isolates belonging to cluster Ib and 25.7% for cluster Ia; however, a higher susceptibility (84.3%) was observed for isolates belonging to cluster II (Table 6).

**Table 5:** Frequency (%) of *Streptococcus agalactiae* isolates (n = 86) belonging to RAPD-clusters (Ia [n = 35], Ib [n = 32], and II [n = 19]) that had 50% (MIC_50_) and 90% (MIC_90_) of bacterial growth inhibited at each antimicrobial concentration. All isolates were identified from clinical mastitis cases occurred in 9 dairy herds of Southeast, Brazil.

Antimicrobial	Cluster	Frequency (%) of isolates at each indicated MIC (μg/mL)^1^												NI^2^	MIC_50_^3^	MIC_90_^4^
		0.12	0.25	0.5	1	2	4	8	16	32	64	128	256			
Ampicillin	Ia	31.4	68.6	0.0	0.0	0.0	0.0	0.0	-	-	-	-	-	-	0.25	0.25
	Ib	100	-	0.0	0.0	0.0	0.0	0.0	-	-	-	-	-	-	0.12	0.12
	II	78.9	15.8	5.3	0.0	0.0	0.0	0.0	-	-	-	-	-	-	0.12	0.25
Ceftiofur	Ia	-	-	71.4	20.0	2.9	2.9	-	-	-	-	-	-	2.9	0.5	1.0
	Ib	-	-	100	0.0	0.0	0.0	-	-	-	-	-	-	-	0.5	0.5
	II	-	-	94.7	5.3	0.0	0.0	-	-	-	-	-	-	-	0.5	0.5
Cephalothin	Ia	-	-	-	-	100	0.0	0.0	0.0	-	-	-	-	-	2.0	2.0
	Ib	-	-	-	-	93.8	3.1	0.0	0.0	-	-	-	-	3.1	2.0	2.0
	II	-	-	-	-	94.7	5.3	0.0	0.0	-	-	-	-	-	2.0	2.0
Erythromycin	Ia	-	97.1	0.0	0.0	0.0	2.9	-	-	-	-	-	-	-	0.25	0.25
	Ib	-	31.2	12.5	12.5	9.4	9.4	-	-	-	-	-	-	25	1.0	>4
	II	-	89.5	5.3	5.2	0.0	0.0	-	-	-	-	-	-	-	0.25	0.50
Oxacillin	Ia	-	-	-	-	100	0.0	-	-	-	-	-	-	-	2.0	2.0
	Ib	-	-	-	-	93.8	0.0	-	-	-	-	-	-	6.2	2.0	2.0
	II	-	-	-	-	94.7	5.3	-	-	-	-	-	-	-	2.0	2.0

^1^The light gray shading represents the susceptible zone, and the darker gray shading represents the resistant zone. Results were interpreted according to the Clinical and Laboratory Standards Institute (CLSI, [28, 29]). Interpretative criteria were based on human data (ampicillin, erythromycin, oxacillin, penicillin, sulfadimethoxine and tetracycline), dogs’ data (cephalothin), and bovine mastitis (ceftiofur, penicillin/novobiocin and pirlimycin). The resistant category included those isolates categorized as either intermediate or resistant.

^2^NI = Not inhibited (growth at highest concentration tested).

^3^MIC (μg/mL) that inhibited 50% (MIC_50_) of the isolates.

^4^MIC (μg/mL) that inhibited 90% (MIC_90_) of the isolates.

**Table 6:** Frequency (%) of *Streptococcus agalactiae* isolates (n = 86) belonging to RAPD-clusters (Ia [n = 35], Ib [n = 32], and II [n = 19]) that had 50% (MIC_50_) and 90% (MIC_90_) of bacterial growth inhibited at each antimicrobial concentration. All isolates were identified from clinical mastitis cases occurred in 9 dairy herds of Southeast, Brazil.

Antimicrobial	Cluster	Frequency (%) of isolates at each indicated MIC (μg/mL)^1^												NI^2^	MIC_50_^3^	MIC_90_^4^
		0.12	0.25	0.5	1	2	4	8	16	32	64	128	256			
Penic+Novob.	Ia	-	-	-	100	0.0	0.0	0.0	-	-	-	-	-	-	1.0	1.0
	Ib	-	-	-	100	0.0	0.0	0.0	-	-	-	-	-	-	1.0	1.0
	II	-	-	-	100	0.0	0.0	0.0	-	-	-	-	-	-	1.0	1.0
Penicillin	Ia	94.3	5.7	0.0	0.0	0.0	0.0	0.0	-	-	-	-	-	-	0.12	0.12
	Ib	100	0.0	0.0	0.0	0.0	0.0	0.0	-	-	-	-	-	-	0.12	0.12
	II	100	0.0	0.0	0.0	0.0	0.0	0.0	-	-	-	-	-	-	0.12	0.12
Pirlimycin	Ia	-	-	100	0.0	0.0	0.0	-	-	-	-	-	-	-	0.5	0.5
	Ib	-	-	53.2	3.1	0.0	3.1	-	-	-	-	-	-	40.6	0.5	>4
	II	-	-	100	0.0	0.0	0.0	-	-	-	-	-	-	-	0.5	0.5
Sulfadimet.	Ia	-	-	-	-	-	-	-	-	17.1	34.3	37.1	8.6	2.9	64	256
	Ib	-	-	-	-	-	-	-	-	34.3	56.3	6.3	3.1	-	64	64
	II	-	-	-	-	-	-	-	-	10.6	52.6	26.3	10.5	-	64	256
Tetracycline	Ia	-	-	-	17.1	8.6	0.0	2.9	-	-	-	-	-	71.4	>8	>8
	Ib	-	-	-	3.1	3.1	3.1	0.0	-	-	-	-	-	90.6	>8	>8
	II	-	-	-	63.2	21.1	5.3	0.0	-	-	-	-	-	10.5	1.0	>8

^1^The light gray shading represents the susceptible zone, and the darker gray shading represents the resistant zone. Results were interpreted according to the Clinical and Laboratory Standards Institute (CLSI, [28, 29]). Interpretative criteria were based on human data (ampicillin, erythromycin, oxacillin, penicillin, sulfadimethoxine and tetracycline), dogs’ data (cephalothin), and bovine mastitis (ceftiofur, penicillin/novobiocin and pirlimycin). The resistant category included those isolates categorized as either intermediate or resistant.

^2^NI = Not inhibited (growth at highest concentration tested).

^3^MIC (μg/mL) that inhibited 50% (MIC_50_) of the isolates.

^4^MIC (μg/mL) that inhibited 90% (MIC_90_) of the isolates.

There were no differences in the values of MIC_50_ and MIC_90_ among RAPD-clusters for cephalothin (2 μg/mL), oxacillin (2 μg/mL), penicillin+novobiocin (1 μg/mL) and penicillin (0.12 μg/mL). However, differences in the values of MIC were observed for the other six antimicrobials evaluated (Table 5 and Table 6). For example, the MIC_90_ of erythromycin for isolates assigned to cluster Ia was 0.25 μg/mL, which is the lowest concentration of this antimicrobial contained in the panel; however, more than 10% of isolates assigned to clusters Ib were not inhibited at the highest antimicrobial concentration contained in the microdilution test (Table 5). For pirlimycin, although 100% of isolates assigned to clusters Ia and II were inhibited at 0.5 μg/mL (the lowest antimicrobial concentration in the test), 40.6% of isolates assigned to cluster Ib were not inhibited at the highest antimicrobial concentration contained in the microdilution test (Table 6).

#### Survival function analysis

**Fig 2:**
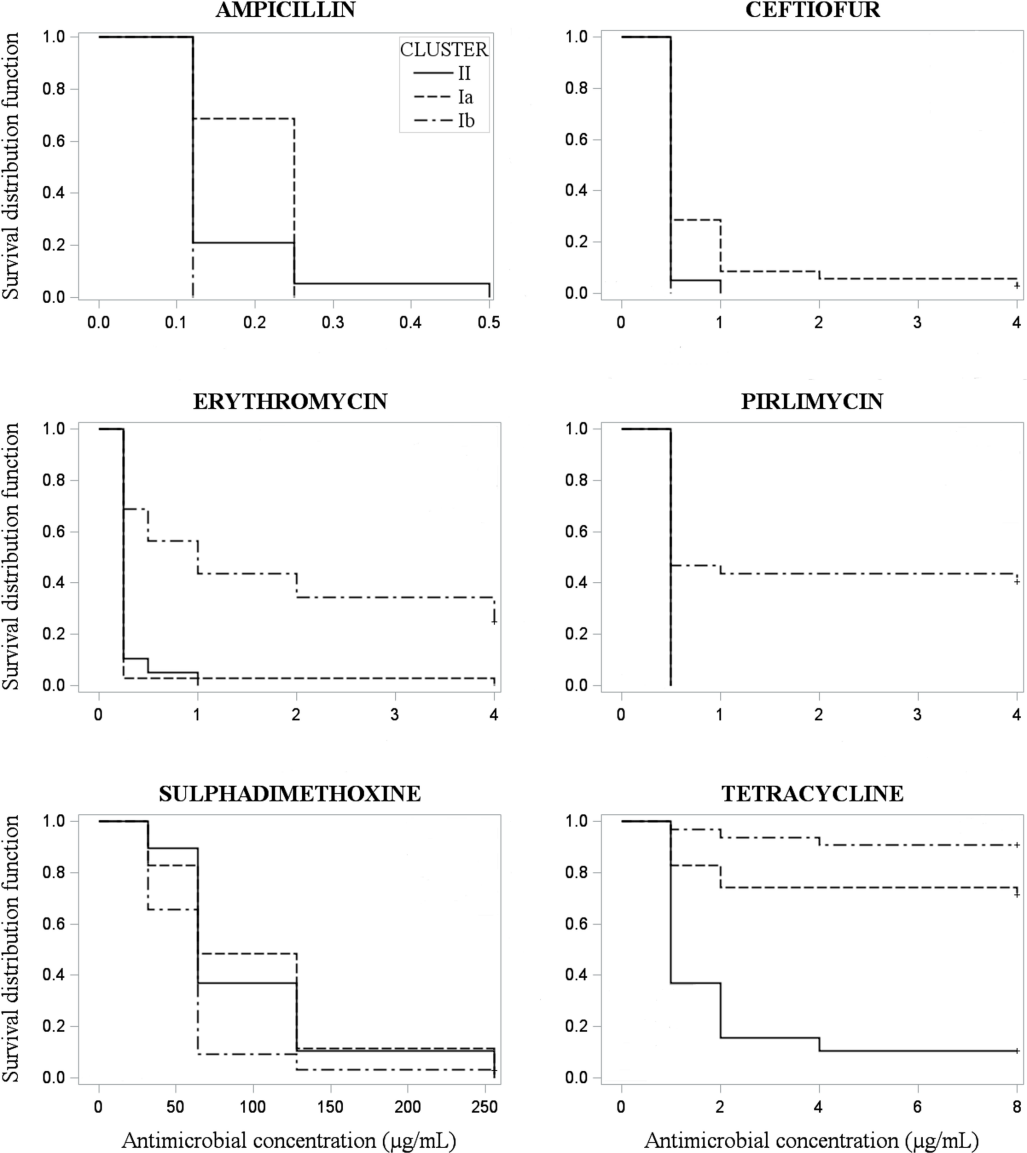
Heterogeneous Kaplan-Meier survival curves (Log-Rank ≤ 0.05; Wilcoxon ≤ 0.0001) of 86 *Streptococcus agalactiae* isolated from clinical mastitis in 9 dairy herds, according to antimicrobial susceptibility testing and stratified by clusters generated based on the genetic similarity of isolates (cluster Ia, n = 35; cluster Ib, n = 32; or cluster II, n = 19).

Kaplan-Meier survival curves were produced for each antimicrobial for comparison of susceptibility among genotypic clusters. Homogeneous survival curves were observed for cephalothin (Log-Rank = 0.30; Wilcoxon = 0.34), oxacillin (Log-Rank = 0.21; Wilcoxon = 0.34), penicillin+novobiocin (Log-Rank = 1.0; Wilcoxon = 1.0), and penicillin (Log-Rank = 0.22; Wilcoxon = 0.22). On the other hand, heterogeneous survival curves among genotypic clusters were obtained for ampicillin (Log-Rank <0.0001; Wilcoxon <0.0001), ceftiofur (Log-Rank = 0.0001; Wilcoxon = 0.0001), erythromycin (Log-Rank <0.0001; Wilcoxon <0.0001), pirlimycin (Log-Rank <0.0001; Wilcoxon <0.0001), sulfadimethoxine (Log-Rank = 0.003; Wilcoxon <0.003), and tetracycline (Log-Rank <0.001; Wilcoxon <0.001; Fig 2).

## Discussion

A great intraspecific diversity (45 RAPD-types) of *Strep*. *agalactiae* causing CM was observed in this study considering the tolerance of 1% in the genetic similarity among isolates. Despite the high level of polymorphism, all isolates shared more than 92% similarity and it may be because more than one strain were selected per herd. A study characterizing *Strep*. *agalactiae* isolated from bovine mastitis and human infections reported 58% similarity among all isolates using the same typing method as used in this study [18]; however, after clustering, the isolates shared 70% genetic similarity.

The high frequency of genetic similarity among *Strep*. *agalactiae* isolates in our study may be due to contagious transmission of this pathogen, which has the bovine udder as the main source of the organism in dairy herds [1]. For all herds, especially those with a greater number of isolates, there was a predominance of strains belonging to the same cluster. For example, all isolates identified in herd N belonged to cluster “Ia”, while 91.7% of isolates from herd Q belonged to cluster “Ib”. These two herds accounted for approximately 63% of all isolates in this study.

Other studies also reported high genetic similarity of *Strep*. *agalactiae* causing IMI within herds, and even within region [14, 16, 21]. Using a multiplex-PCR assay for capsular polysaccharide (CPS) typing, a Brazilian study reported great similarity between *Strep agalactiae* isolates within the geographical region from which the isolates were identified [16]. Another molecular study evaluating six Danish dairy herds with high prevalence of *Strep*. *agalactiae* reported the predominance of a single PFGE-type and sequence type (ST) within herd [14]. Moreover, Rato et al. [13] genotyped 60 *Strep*. *agalactiae* isolates from 6 dairy herds in Portugal and found four major clonal clusters sharing ≥80% similarity among strains.

The within-herd predominance of *Strep*. *agalactiae* strains, or even clusters composed of closely-related strains, is consistent with the contagious transmission of this pathogen. *Streptococcus agalactiae* is mainly transmitted because of inadequate milking routine in the milking parlor, which increases the risk of a healthy cow coming into contact with equipment, hands or towels contaminated by milk from an infected cow [1]. Therefore, management practices aiming to reduce the contact of healthy quarters with contaminated milk, such as adequate milking routine, use of disposable gloves, use of post milking teat disinfectants, and treatment and segregation of infected cows, are recommended for the reduction of new IMI caused by *Strep*. *agalactiae* in dairy herds.

In our study, three herds (I, M and Q) had strains assigned to more than one cluster, which indicate the presence of strains with genetic differences within herds. Considering that infected cows are the primary reservoir of *Strep*. *agalactiae*, it would be expected that all strains within herds belonged to the same cluster. However, the presence of strains with different genotypic characteristics within herd may be attributed to the lack of biosecurity programs, especially due to the purchase of cows with *Strep*. *agalactiae* infection. Therefore, assessment should take place at the herd of origin before purchasing cows, which should include the SCC history for the cow, records of CM and previous culture information. If this prepurchase information is not available, milk cultures should be conducted shortly after arrival and the cow should be considered potentially infected until results are available [1].

In addition to biosecurity issues, the presence of isolates with different genotypic characteristics within herds could be attributed to different reservoirs of *Strep*. *agalactiae* other than infected mammary gland. A recent study reported that is also possible that different *Strep*. *agalactiae* strains associated with bovine mastitis favor different niches and transmission routes, as this pathogen was isolated from the rectum and environment of dairy cows [32]. Moreover, current control measures of *Strep*. *agalactiae* in dairy herds may also take into account the fecal and environmental reservoirs of this pathogen.

Differences in the frequency of strains within clusters among housing system were also observed in our study. Approximately 80% of isolates identified in herds housing cows in free stalls belonged to cluster Ia, while 65% of isolates identified in CBPB were assigned to cluster Ib. Moreover, 4 of 5 isolates identified from herds housing cows in paddocks belonged to cluster II. However, because *Strep*. *agalactiae* is mainly transmitted during milking, it is likely that the similarity between isolates within housing system is more associated with other risk factors at the herd level, such as management practices related to the milking procedure and within-herd biosecurity, than with the type of housing facility.

Interesting results were also observed in the evaluation of AMS of *Strep*. *agalactiae* isolates. Eight of 10 antimicrobials tested had the MIC_50_ at the lowest antimicrobial concentration contained in the microdilution test. High AMS was seen mainly for β-lactams antimicrobials. On the other hand, *Strep*. *agalactiae* isolates were less susceptible to other antimicrobials such as tetracycline (31.4%) and erythromycin (70.9%).

The overall resistance to erythromycin in our study was 29.1%, although difference in the susceptibility were observed among clusters. Comparison of our results and other studies evaluating AMS should be done with caution because of differences in techniques used (disk-diffusion or broth dilution test), as well as differences of interpretative criteria used for clinical categorization of isolates as resistant or susceptible. However, resistance to erythromycin was also reported for isolates identified from bovine mastitis [25, 33], and clinical isolates recovered from human diseases [34]. A Korean study evaluating 185 human clinical *Strep*. *agalactiae* isolates reported an increase of erythromycin resistance from zero in 1990 to 40% in 1998 [34]. Furthermore, Dogan et al. [25] reported 27% of resistance to erythromycin in *Strep*. *agalactiae* recovered from human diseases, but a lower frequency of resistance was found for isolates recovered from bovine mastitis (3.6%). In general, the resistance of *Strep*. *agalactiae* to erythromycin seems to be lower in cattle than human isolates, ranging from zero in a French study [35] to 10.5% in a Brazilian report [36].

Our study showed significant differences of AMS to erythromycin and pirlimycin among clusters, and these results were well illustrated in the Kaplan-Meier survival curves. Isolates assigned to cluster Ib were inhibited at higher erythromycin and pirlimycin concentrations than isolates belonging to clusters Ia and II. Duarte et al. [36], evaluating isolates recovered from milk of dairy cows with clinical or subclinical mastitis reported that resistance to erythromycin was predominantly associated with isolates belonging to serotype II (50%) and III (22.2%). Likewise, resistance to pirlimycin was reported for *Streptococcus* spp. (including *Strep*. *agalactiae*) isolated from subclinical mastitis, which was described to be associated to the presence of *lin*(B) gene that encodes the resistance to lincosamides [13]. Therefore, the variation of the AMS of *Strep agalactiae* between studies, and among different genotypes (or clusters) within studies, may be explained by genetic mechanisms of resistance. Besides the *linB*, other genes affecting the resistance to macrolides and lincosamides, such as *lnu*(D), *emr*(A) and *emr*(B), were associated with the antimicrobial resistance of isolates recovered from clinical and subclinical mastitis in dairy cattle [22, 36]. Therefore, it is reasonable to infer that isolates belonging to cluster Ib in our study may have genetic determinants associated with resistance to erythromycin and pirlimycin.

In relation to tetracycline, our results showed 70% of *Strep*. *agalactiae* resistance and heterogeneous curves were observed in the survival analysis comparing the AMS among clusters. A total of 84.2% of isolates belonging to cluster II were susceptible to tetracycline, while only 25.7% of isolates pertaining to cluster Ia, and 6.3% of isolates assigned to cluster Ib were susceptible to this antimicrobial. Likewise, high prevalence of *Strep*. *agalactiae* resistant to tetracycline, as well as differences in the susceptibility among genotypes, were reported in other studies. Tetracycline resistance was described for 44.7% of isolates in a study evaluating *Strep*. *agalactiae* recovered from dairy cattle in Brazil [36], and similar results (44.7%) were observed in a study performed in Poland [22]. Moreover, a study conducted in Portugal reported approximately 65% of tetracycline resistance in *Strep*. *agalactiae* isolates (n = 60) recovered from subclinical mastitis in dairy cows [13].

The low efficacy of tetracycline against *Strep*. *agalactiae* has been attributed to the excessive use of this antimicrobial in the past, especially as prophylactic agents or as a growth promoter [13, 37]. Furthermore, several tetracycline resistance-related genes were identified in *Strep*. *agalactiae* isolated from humans and bovine IMI. Most tetracycline-resistant strains characterized in a Polish study had at least one of the five evaluated resistance genes (*tet*(O), *tet*(L), *tet*(M), *tet*(K) and *tet*(S)) [22]. Another study evaluating the resistance genes among *Strep*. *agalactiae* isolates recovered from bovine mastitis and human diseases also reported high frequencies of genes associated with tetracycline resistance [33]. Therefore, considering that resistance genes can either be present or absent in specific genotypes, we could infer that the differences in the tetracycline susceptibility among clusters in our study could be associated with different frequency of resistance genes among isolates. However, although we found that cluster II had a relatively high proportion of isolates susceptible to tetracycline, this antimicrobial would not be the first choice for treatment of CM caused by *Strep*. *agalactiae* due to the high overall antimicrobial resistance observed in the current study and other reports [33].

In contrast, β-lactams antimicrobials are still the first choice for treatment of infections caused by *Streptococcus* spp., and our results on AMS of *Strep*. *agalactiae* are in agreement with these findings. In our study, the MIC_90_ of β-lactams antimicrobials was determined at the lowest or second lowest antimicrobial concentration present in the test, which indicates the high efficacy of these antimicrobials against *Strep*. *agalactiae in vitro*. This low β-lactams resistance of *Strep*. *agalactiae* isolates found in our study agrees with the results reported elsewhere [22, 35].

Although there was high susceptibility to all antimicrobials pertaining to β-lactams class, heterogeneous survival curves were observed among clusters in our study for ampicillin and ceftiofur. These results suggest that some intraspecific genetic determinants may be involved with *Strep*. *agalactiae* susceptibility to these antimicrobials. Therefore, further studies evaluating the genomic characteristics of *Strep*. *agalactiae* strains should be conducted to determine the distribution of antimicrobial resistance genes, which could identify genetic differences associated with AMS among genotypic clusters found in this study.

## Conclusion

High genotypic diversity was found among *Strep*. *agalactiae* isolates; however, after clustering based on RAPD results, a high genotypic similarity was observed within and between herds. Overall, *Strep*. *agalactiae* genotypes were highly susceptible to most evaluated antimicrobials, except to erythromycin and tetracycline. Finally, differences in the survival curves were observed among genotypic clusters for six (ampicillin, ceftiofur, erythromycin, pirlimycin, sulfadimethoxine and tetracycline) of 10 antimicrobials evaluated, indicating that AMS can vary according to the strain of *Strep*. *agalactiae* causing CM. Next steps in our research include assessing potential genetic characteristics (e.g., antimicrobial resistance properties) among *Strep*. *agalactiae* isolates that could help us to further understand the differences in AMS observed in the present study. In addition, it may be worth to conduct an epidemiological study to evaluate the association of herd-level factors (e.g., biosecurity practices) associated with the presence of different *Strep*. *agalactiae* strains within herds; as well as, the assessment of potential reservoirs of *Strep*. *agalactiae* in dairy herds other than the infected mammary gland.
